# Spontaneous Remission After a Hypercalcemic Crisis Caused by an Intracystic Hemorrhage of Bilateral Parathyroid Adenomas: A Case Report and Literature Review

**DOI:** 10.3389/fendo.2021.766234

**Published:** 2021-10-25

**Authors:** Yaoxia Liu, Jianwei Li, Hui Liu, Han Yang, Jingtao Qiao, Tao Wei, Tao Wang, Yerong Yu

**Affiliations:** ^1^ Department of Endocrinology and Metabolism, West China School of Medicine/West China Hospital of Sichuan University, Chengdu, China; ^2^ Department of Geriatrics, Sichuan Provincial People’s Hospital, University of Electronic Science and Technology of China, Chengdu, China; ^3^ Department of Geriatrics, Chinese Academy of Sciences Sichuan Translational Medicine Research Hospital, Chengdu, China; ^4^ Department of Thyroid Surgery, West China School of Medicine/West China Hospital of Sichuan University, Chengdu, China; ^5^ Department of Pediatrics, West China Second University Hospital, Sichuan University, Chengdu, China; ^6^ The Cardiac Development and Early Intervention Unit, West China Institute of Women and Children’s Health, West China Second University Hospital, Sichuan University, Chengdu, China; ^7^ Key Laboratory of Birth Defects and Related Diseases of Women and Children (Sichuan University), Ministry of Education, Chengdu, China

**Keywords:** hypercalcemic crisis, spontaneous remission, hemorrhage, parathyroid adenoma, primary hyperparathyroidism

## Abstract

**Background:**

Hyperparathyroidism is a common cause of hypercalcemia; however, spontaneous remission after a hypercalcemic crisis caused by an intracystic hemorrhage of parathyroid adenomas is very rare. The question, then, is “What is the best treatment strategy for this type of case?”

**Method:**

A 47-year-old male patient with primary hyperparathyroidism and a hypercalcemic crisis is reported. Hypercalcemia was spontaneously relieved thereafter. Postoperative paraffin pathology results indicated an intracystic hemorrhage of bilateral parathyroid adenomas.

**Results:**

After the case report, a literature review is also included to summarize the clinical features of this patient and to provide special reference for clinical diagnosis and treatment of similar cases.

**Conclusions:**

The choice of surgical timing for such cases can be made based on the comprehensive consideration of clinical symptoms and changes in parathyroid function.

## Introduction

Hyperparathyroidism is the most common cause of hypercalcemia, but spontaneous remission after a hypercalcemic crisis caused by hemorrhage of parathyroid adenomas is very rare. There have been no reports involving an intracystic hemorrhage of bilateral parathyroid adenomas. It is generally believed that hemorrhage of parathyroid adenomas may be caused by rapid tumor growth with an insufficient blood supply to the tumor (1). The patients may present with a diversity of clinical features due to differing degrees of hemorrhage and necrosis of the parathyroid adenoma cells (2). Indeed, there has not been a literature review on the appropriate clinical decision for spontaneous remission of parathyroid function following an intracystic hemorrhage of parathyroid adenomas.

## Case Description

**Figure 1:**
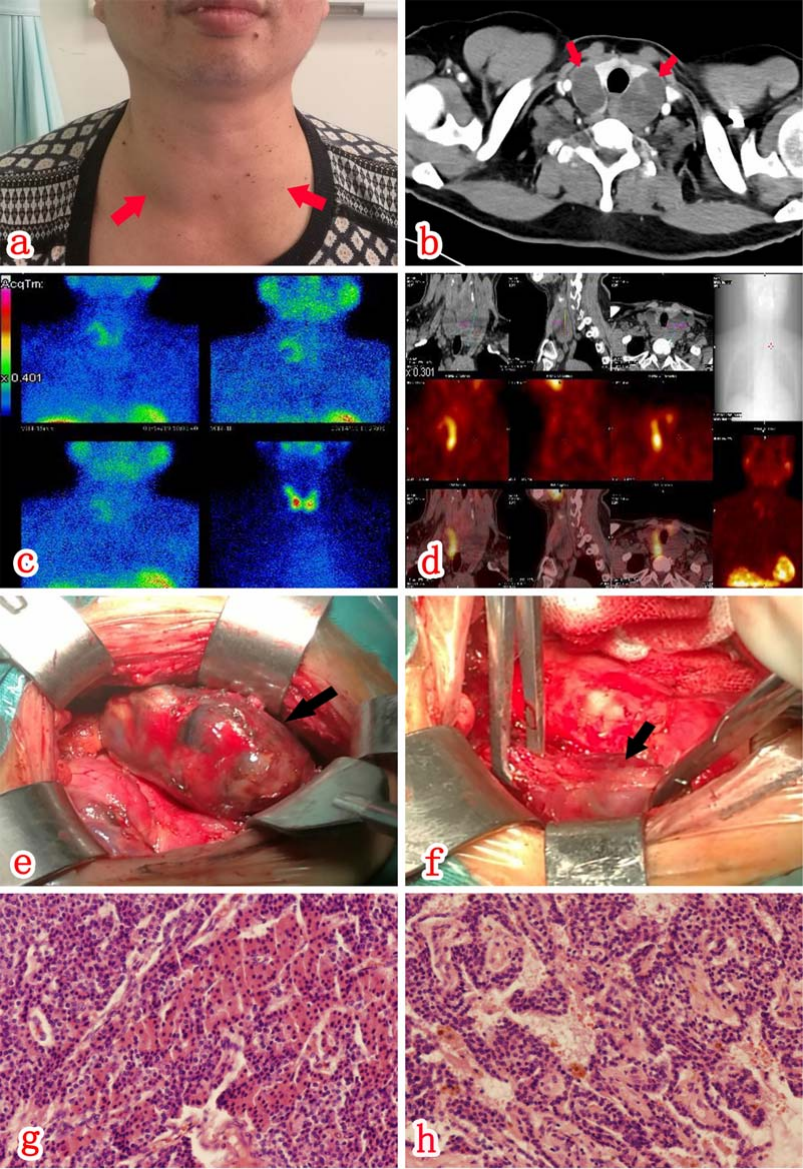
There was a grade II goiter **(A)**, with a 4 x 4 cm mass palpable near the thyroid bilaterally. CT scan **(A, B)** of the neck revealed bilateral slightly hypointense cystic shadows below the thyroid. SPECT/CT fusion imaging **(C, D)** revealed an abnormal increase in uptake in part of the mass behind the right lobe of the thyroid during MIBI, which was considered relevant to hyperparathyroidism. There were a cystic-solid mass **(E)** on the posterolateral aspect of the right lateral lobe of the thyroid and a cystic-solid mass **(F)** on the left lateral lobe of the thyroid. Postoperative paraffin pathology (HE staining x100) results indicated that both the right **(G)** and left **(H)** space-occupying lesions represented parathyroid adenomas with cystic change.

A 47-year-old male patient was referred to onset of excessive thirst and polydipsia more than 1 month ago, accompanied by general malaise and anorexia. Two weeks ago he went to a local hospital due to abdominal discomfort and the blood biochemical test revealed both elevated serum creatinine (172.9 umol/L,Ref. 63-109 umol/L) and calcium level(4.66 mmol/l, Ref. 2.1-2.7 mmol/L), a declined phosphorus level(1.76 mmol/l, Ref. 0.81-1.45 mmol/L). The blood sodium, potassium, and glucose levels were all normal. The patient was then referred to our hospital for further evaluation. The patient was previously healthy, without psycho-social disease,trauma history, nor drug use. He also denied the family history of hypercalcemia. Physical examination revealed the following: T, 36.5°C; P, 91/min; R, 20/min; and BP, 139/96 mmHg. No abnormalities were detected on examination of the heart, lungs, and abdomen. There was a grade II goiter (**Figure 1A**), with a 4 x 4 cm mass palpable in front of the neck. The masses had hard texture with poor mobility and no tenderness. The results of blood biochemical testing on the day after admission were as follows: creatinine 195 umol/L; estimated glomerular filtration rate 34.3 ml/min/1.73m^2^; calcium 3.80 mmol/L; phosphorus 1.24 mmol/L; and PTH, 320.7 pmol/L (Ref. 1.6-6.9 pmol/L).

**Figure 2:**
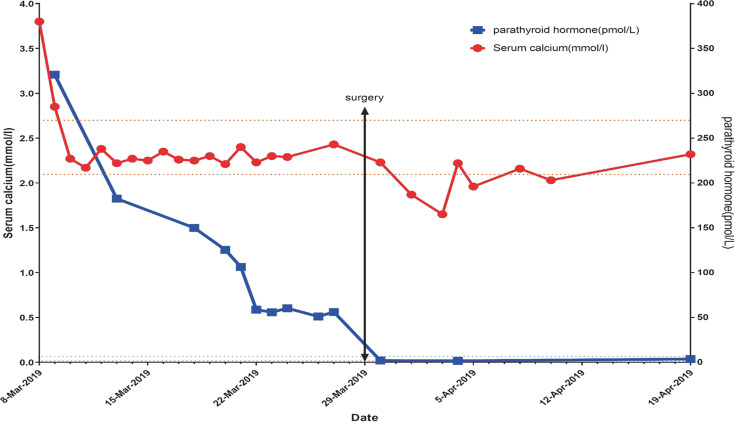
After admission, the blood calcium level was 3.6 mmol/L. After 2000-4000 ml of NS for 5 days and salmon calcitonin (300 IU ivgtt), the blood calcium level gradually decreased and remained within the normal range. After surgery, the blood calcium level decreased, reaching the lowest level (1.65 mmol/L). After that, the calcium level was gradually restored to normal. There was a progressive decrease in the PTH level; however, the PTH level was stabilized at 50-60 pmol/L at 12 days post-admission, and this situation persisted for 1 week. One day post-operatively the PTH level rapidly decreased to 1.95 pmol/L, followed by a slow, gradual increase.

The patient was given isotonic saline infusion (200 to 300 mL/hour) to correct volume depletion, a loop diuretic [furosemide (20 mg iv qd for 2 days)] to increase calcium excretion, and a single dose of salmon calcitonin (300 IU ivgtt). Third day after admission, the patient’s serum calcium level decreased to 2.17 mmol/l and the PTH level was 182.5 pmol/L. Then rehydration and calcium-lowering therapy were stopped, and the serum calcium concentration was monitored every one to two days. During the next one week the serum calcium levels had been within the normal range and phosphorus level fluctuated between 0.36 and 0.50 mmol/L. The serum creatinine levels returned to normal and PTH gradually decreased, but still above normal (**Figure 2**).

A CT scan of the neck (**Figure 1B**) revealed bilateral slightly hypointense cystic mass deep the thyroid, 4.4 x 3.2 cm size on the right, 4.2 x 2.1 cm size on the left, with a uniform density and clear borders. A contrast-enhanced scan revealed intracystic septa within the lesions, with apparent enhancement of the cystic wall and septa. An ultrasound examination of the neck revealed mixed cystic-solid nodules posterior to the two lateral lobes of the thyroid, the size was 5.0 x 2.3 x 3.7 cm(right) and 5.3×3.5×3.1cm(left) respectively. Both nodules had clear borders and a regular morphology without apparent internal blood flow signals. Contrast-enhanced ultrasound of neck mass shows mixed cystic-solid nodules behind the two lateral lobes of the thyroid. SPECT/CT fusion imaging (MIBI; **Figures 1C, D**) revealed an abnormal increase uptake during MIBI in part of the mass posterior to the right lobe of the thyroid, which point the possibility of parathyroid adenoma. A bone density test and X-ray examination of the hands and four limbs did not demonstrate a reduction in bone mass and osteoporosis. At 11th day post-admission, the patient underwent an ultrasound-guided biopsy of the bilateral cystic nodules posterior to the thyroid. Ten milliliters of dark red bloody fluid were collected from the cystic nodules bilaterally. Exfoliative cytology indicated a small number of hyperplastic epithelial and tissue cells, possibly indicating benign cystic lesions. The neck masses shrank in size after fine needle aspiration; however, ultrasonography of the thyroid gland 2 days later revealed cystic space regains its size before aspiration which suggested new hemorrhage after the fine needle aspiration.

According to the guidelines for the diagnosis and treatment of primary hyperparathyroidism (3) and considering the patient’s surgical requirements, the neck mass resection was performed at three weeks post-admission, during which the bilateral cystic-solid masses were found, 5 x 3 x 3.5 cm in size on the right (**Figure 1E**) and 5.5 x 4 x 3.5 cm on the left (**Figure 1F**). The mass adhered closely to the thyroid gland and the surrounding tissues, with obscure borders and an irregular morphology. At last, the patient underwent resection of bilateral inferior parathyroid adenomas and a total thyroidectomy. Bilateral superior parathyroids were preserved. Postoperative paraffin pathology results indicated intracystic hemorrhage of bilateral parathyroid adenomas. (**Figures 1G, H**). Thyroid pathology suggested nodular goiter in both bilateral lobes and isthmus of the thyroid gland.

The patient recovered well after surgery. At day 1 post-operatively, the serum PTH level was 1.95 pmol/L, and the blood calcium level was 2.23 mmol/L. According the clinical guideline, The patient was given calcitriol [1,25(OH)2D] 0.5ug twice daily, calcium supplement (10% calcium gluconate 100ml diluted intravenous infusion each day) after surgery. At 2 and 4 days post-operatively, the blood calcium level was 1.87 and 1.65 mmol/L, respectively, and the patient had no tetany. At 9 days post-operatively, the normal blood calcium level was restored and discharged from hospital with oral calcium carbonate 600mg twice daily and osteopontin 0.5ug twice daily. The blood calcium and PTH levels were monitored at 3 weeks, 2 months, 4 months, post-operatively and the results were all normal. The dose of the above drugs was reduced at month 4 and then discontinued at month 5. Calcium and phosphorus were still normal at the 6th month recheck.

Through screening tests,the patient was finally ruled out multiple endocrine neoplasia. He and his relatives also underwent testing of genes associated with MEN, none were carriers of the MEN related genes.

## Discussion

### The Mismatch Between Tumor Rapid Growth and Blood Supply Might Result in Infarction of Parathyroid Adenoma and Hemorrhage

Intracystic hemorrhage of parathyroid adenomas is very rare. There are only about 100 cases reported with intracystic hemorrhage of parathyroid adenomas in the literatures. Some studies have shown that the use of anti-coagulation drugs (4) non-steroidal anti-inflammatory drugs (5) and trauma (6) may be risk factors; however, the cause of intracystic hemorrhage of parathyroid adenomas is unknown. It is currently believed that if tumor growth is too rapid and the blood supply to the tumor is insufficient, hemorrhage of parathyroid adenomas may occur (1). In 1953, Howard reported spontaneous remission after hyperparathyroidism due to infarction in parathyroid adenomas (7), which was called Parathyroidectomy. Nylen (8) suggested that infarction and hemorrhage represented two stages of the same phenomenon. Parathyroid adenoma apoplexy is divided into infarction of the adenoma (without hemorrhage), infarction with intracystic hemorrhage, and extracystic hemorrhage (8).

For our patient, he was in good health before and without trauma nor drug use history. Combined with the changes of blood calcium level, it is speculated that the rapid growth of adenoma and the massive release of PTH lead to the hypercalcemic crisis. However, the mismatch between tumor growth and blood supply resulted in infarction of parathyroid adenoma and intracystic hemorrhage, followed by spontaneous remission of hyperparathyroidism and hypercalcium crisis.

### The Clinical Manifestations of Bleeding Parathyroid Adenomas and the Accompanying Changes in Parathyroid Function Are Diverse

Symptoms of hemorrhage of parathyroid adenomas depend on the speed and volume of hemorrhage, as well as the position of the parathyroid gland (*in situ* or ectopic). Simple necrosis of parathyroid adenomas is associated with a smaller hematoma and few local symptoms. This condition may remain undetected until an incidental finding of spontaneous remission of hypercalcemia (9). Some patients are free from local discomfort in intracystic hemorrhage of *in situ* parathyroid adenomas (2), and may present with neck pain, a mass, and hoarseness (2). In *in situ* extracystic hemorrhage, the blood may diffuse to the neck or mediastinum, leading to compression of tracheal and esophagus (2), may cause dyspnea and dysphagia. Compression of the recurrent laryngeal nerve will lead to hoarseness and vocal cord paralysis with local swelling, pain, a mass, and ecchymosis (2) Spread of the hematoma may cause a pleural effusion (10, 11). Life-threatening hemorrhage of parathyroid adenomas usually needs surgical treatment as soon as possible. Hemorrhage of ectopic parathyroid adenomas often leads to a mediastinal hematoma, (6, 11–16) which also requires emergency surgery.

Parathyroid adenoma cells may also be involved and become necrotic after necrosis and hemorrhage of parathyroid adenomas, so parathyroid function will be influenced too. The changes of parathyroid function vary to what extent the adenoma cells are involved. Some patients with hypercalcemia fail to achieve remission (17–19) or even progress to refractory hypercalcemic crisis (20). Others with hypercalcemia may show a reduction in the PTH level and achieve remission or even have the blood calcium level restored to normal (7, 21). In a few cases, hungry bone syndrome may occur due to the rapid decrease in the PTH level, leading to hypocalcemia (2, 22–24). Patients with hyperparathyroidism and complete necrosis of the parathyroid adenoma usually achieve full remission (21, 24–28), i.e., parathyroid adenomectomy in the real sense; however, some patients with hypercalcemia only achieve temporary remission and relapse later (22, 24, 27, 29). The time of relapse varies from one patient to another and ranges from several weeks to several years. The longest reported time of relapse after remission is 7 years (30).

In our case, only bilateral thyroid mass was present because of bilateral *in situ* parathyroid adenoma intracystic hemorrhage. Bone X-ray and bone density testing did not reveal bone mass changes in our case, indicating a relatively short course of disease of hyperparathyroidism. This patient showed a significant increase in the PTH level upon the onset, indicating rapid growth of the parathyroid adenoma and the secretion of a large amount of hormone before the hemorrhage. The blood calcium level was restored spontaneously to normal after the hypercalcemic crisis; however, there was no further decrease in the PTH level beyond 50-60 pmol/L. Based on an increased uptake in MIBI, it was concluded that there were residual parathyroid adenoma cells. The PTH level of this patient was still above the normal range, while the blood calcium level was low, which was possibly due to the decrease in PTH receptor sensitivity under a high PTH level. Considering the possibility of recurrent hypercalcemia and the surgical requirement of the patient, and according to the guidelines (3) this patient finally received surgical treatment. The post-operative pathologic results confirmed intracystic hemorrhage of bilateral parathyroid adenomas.

### Fine-Needle Aspiration Biopsy of Parathyroid Adenoma Hematoma Is of Limited Diagnostic Value and Carries Some Risk

In our patient, fine needle aspiration was performed to determine the etiology of bilateral parathyroid cystic lesions and collected bloody fluid eventually. Pathologic evaluation results showed a small amount of hyperplastic epithelial and tissue cells. After the fine needle aspiration biopsy, the cystic space-occupying lesions shrank in size, but soon grew to the original size. We further reviewed the relevant literature to explore the value and risks of fine-needle aspiration biopsy in such cases.

There have been previous reports on fine needle aspiration biopsy for hemorrhage of parathyroid adenomas. Givens (31) reported one case with hemorrhage of parathyroid adenoma who underwent a CT-guided biopsy. Pathologic biopsy showed epithelioid cells in well-ordered sheets and rare atypical cells. Other studies (8, 32) described bloody fluid from fine needle biopsy aspiration, which confirmed hemorrhage, but without a pathologic diagnosis. Novodvorsky (24) and Taguchi (33) reported that the finding of an increase in PTH level in the puncture fluid could help establish the diagnosis of parathyroid adenomas. Simcic (34) reported decompression of the hematoma by fine needle aspiration. The patient had immediate relief of his discomfort, and the mass regressed considerably in size, only to recur within 24 h of the puncture. This situation was similar to that observed in our patient. But Shim report that for patients with neck swelling and pain due to hemorrhage of the parathyroid adenoma, conservative management with intermittent sono-guided aspiration was performed without specific events (35).

The finding of bloody fluid by fine needle aspiration biopsy and an increase in PTH level in puncture fluid can help to make the diagnosis of hemorrhage of parathyroid mass and hyperparathyroidism for patients with cystic space-occupying lesions in the parathyroid gland, but it is difficult to obtain valuable pathologic cells in the puncture fluid. Although decompression of hematoma by fine needle aspiration is conducive to the remission of compression, the risk of another hemorrhage and recurrent hematoma exists.

In addition, there are two case reports on hemorrhage of parathyroid adenomas after fine needle aspiration biopsy so far. One of them was followed up for 105 months, and finally achieved full remission of hyperparathyroidism after hemorrhage of the parathyroid adenoma (25), the other case received surgical treatment at 1 month after aspiration-reduced hemorrhage (36).

Therefore, we believe that fine-needle aspiration biopsy of parathyroid adenoma hematoma is of limited diagnostic value and carries some risk. For patients requiring fine needle aspiration for decompression, the decision should be made carefully by weighing the pros and cons, unless the compression symptoms are severe and urgent.

### Through Literature Review, It Is Believed That There Is No Consensus On the Timing of Surgery for Intracapsular Hemorrhage in Primary *In Situ* Parathyroid Adenomas and That a Comprehensive Assessment Should be Made Based on the Clinical Features of the Case and the Patient’s Wishes

In our case, it was found that the parathyroid adenoma adhered closely to the surrounding tissues and the hematoma tension was high. Both of the hematomas burst open during the surgery. Considering the hyperparathyroidism may relieve after intracystic hemorrhage of parathyroid adenoma and the difficulty of surgery in the acute phase, as well as the risk of extracapsular hemorrhage due to hematoma rupture, we conducted a literature review to answer the following questions. Do these patients require aggressive surgery? What is the appropriate time for surgery?

There has been no consensus on the choice of surgical timing for intracystic hemorrhage of primary *in situ* parathyroid adenomas. According to previous literature reports, some cases had severe adhesions following intracystic hemorrhage of the parathyroid adenomas, as noted during surgery (6, 8, 37). One such case underwent surgery 7 months after the onset (38). The evidence is still insufficient to prove that prolonging conservative treatment can reduce adhesions found by subsequent surgery. In another report (37), a parathyroid adenoma (6.9 cm x 5.2 cm x 4.8 cm) ruptured during surgery. A large amount of unclear and bloody fluid flowed out, as was observed in our case; however, there have been no case reports on extracystic hemorrhage of a parathyroid adenoma due to hematoma rupture during the conservative treatment for intracystic hemorrhage of parathyroid adenomas.

Whether the patients underwent surgery after intracystic hemorrhage of parathyroid adenomas, and the specific duration of conservative treatment vary across the literature reports. We reviewed literature reports on 120 cases with hemorrhage or necrosis of parathyroid adenomas (including primary and secondary hyperparathyroidism, *in situ* ectopic parathyroid adenomas, and intracystic and extracystic hemorrhage of the parathyroid adenomas). Among the cases, we performed a detailed analysis of 34 cases with clinical or pathologic diagnosis of intracystic hemorrhage or necrosis of *in situ* primary parathyroid adenomas, parathyroid cysts and hyperplasia, described in Chinese or English (**Tables 1**, **2**). Of these cases, 12 did not undergo surgical treatment and all of them achieved spontaneous remission of hyperparathyroidism(**Table 1**). Five patients achieved full restoration of parathyroid function and complete absorption of the lesions. Four patients were followed up for 6, 9, 16, and 29 months, and none relapsed. Three patients relapsed at 16 days, 1 year, and 2 years afterwards, although the patients had not undergone surgical treatment upon the time of the literature report. Eight patients finally underwent surgical treatment after conservative treatment. Among the 8 patients, 4 achieved spontaneous remission of hyperparathyroidism, but later underwent surgical treatment due to recurrent hyperparathyroidism at 1 month, 6 years, 20 months, 11 months, and 8 years later. One patient underwent surgery at 2 months after conservative treatment due to failure to achieve remission of hyperparathyroidism; 2 patients achieved remission of hyperparathyroidism to a certain degree, but still received surgical treatment after 1 month and 3 months of conservative treatment, respectively. There were some patients who directly received surgical treatment, but the duration of conservative treatment before surgery was not mentioned in the literature. All patients had their parathyroid adenomas resected en bloc and achieved full remission of hyperparathyroidism after surgery. Whether hyper parathyroidism can be relieved, and hyperparathyroidism will recur later are independent of the size of the hematoma.

**Table 1:** Case analysis of intracystic hemorrhage or necrosis of *in situ* primary parathyroid adenomas (Unoperated patients, n = 12).

Author	Age	Sex	Signs/symptoms	Hematoma size (cm)	[Ca++]	PTH	Surgery	Follow-up time	Changes in PHPT	Clinic Diagnosis
Wootten (2)	63	M	tetanic contractures of the hands	N/A	↓9.4 mg/dL	↑69pg/mL	N	6M	Spontaneous remission	spontaneously resolving primary hyperparathyroidism
Nylen (8)	64	M	Abrupt neck swelling and pain, neck tenderness	1.58	8.6mg/dl	↓27pg/ml	N	16M	Spontaneous remission	parathyroid apoplexy.
Baskar (9)	30	F	Chronic symptomatic hypercalcemia	N/A	→ 2.37mmol/L^†^	↑255ng/L	N	5Y	Spontaneous remission	MEN1
Ferrari (21)	48	M	symptoms of sever symptomatic hypercalcemia	2.8	↑18 mg/dL	↑1315pg/mL	N	2Y	Spontaneous remission	parathyroid apoplexy
Micale Sara J (23)	71	F	neck discomfort, sore throat, difficulty swallowing	2.1 × 2.4 × 3.6	↓8.1 mg/dL	→64.3pg/m	N	16D	Spontaneous remission	infarction of parathyroid adenoma
Novodvorsky (24)	54	F	symptomatic hypocalcaemia	4.4	↓1.88 mmol/L	↑17.6 pmol/L	N	11M	Spontaneous remission	Infarct hemorrhage of parathyroid adenoma
Kara (25)	67	F	slight neck swelling	N/A	→9.3mg/dl	↑90.1	N	105M	Spontaneous remission	hemorrhage of parathyroid adenoma
Schinner (26)	68	M	Chronic symptomatic hypercalcemia	3.7×1.2×1.7	↓3.3 mmol / l	↑9.7 pmol/ l	N	4Y	Spontaneous remission	infarction of the parathyroid adenoma
Onoda (28)	67	F	Asymptomatic	1.4X1.1X1.0	→	→	N	2Y	Spontaneous remission	parathyroid infarction or homeorrhagic infarction
Lucas (29)	53	F	Acute neck pain, dysphagia, neck mass dyspnea	3	→8.6mg/dl	→38pg/ml	N	10M	spontaneous remission, but recurrence in 10 months	infarction of parathyroid adenoma
Kovacs (39)	49	F	Abrupt neck pain,dyspnea,tenderness,	N/A	→ 2.17mmol/L (2.1-2.6)*	↑6.41 (1.38-5.72) pmol/L	N	29M	Spontaneous remission	infarction of the parathyroid adenoma
Chan (40)	78	F	Chronic symptomatic hypercalcemia	2.5 × 1.5	↓ 1.37mmol/L (2.20–2.62 mmol/l)^†^	↑32.5 pmol/l	N	1Y	Spontaneous remission, but recurrence in 1 year	Parathyroid apoplexy

*total calcium; ^†^Corrected calcium; PTH, parathyroid hormone; ↓Below normal; ↑Above normal; →Within the normal range.

**Table 2:** Case analysis of intracystic hemorrhage or necrosis of *in situ* primary parathyroid adenomas (Operated patients, n = 22).

Author	Age	Sex	Signs/symptoms	Hematoma size (cm)	[Ca++]	PTH	Surgery	Time before operation	Changes in PHPT	Pathological diagnosis
Howard (7)	57	F	neck pain, nausea, vomiting and tachycardia supervened	3.5×2×2	↑20 mg. per 100 ml	N/A	surgery	N/A	Spontaneous remission after Hypercalcemia	infarction of the adenoma
DeGroote (17)	45	F	symptomatic hypercalcemia, suddenly neck pain, neck tenderness, Severe dehydration	3x3	20 mg/100 ml	N/A	Neck exploration	4D	N/A	parathyroid adenoma, chief cell type, with a fresh hemorrhagic 1.5-cm cystic area and multiple areas of cystic necrosis within the gland
Chodack (18)	61	F	symptomatic hypercalcemia, gradual increase in sleepiness and confused, neck nontender mass, Tracheal displacement, fever	6.5x4	↑18.5 mg/100 cc	N/A	emergency exploration	emergency exploration	N/A	chief-cell parathyroid adenoma
Mizunashi (20)	62	F	progressive symptomatic hypercalcemia.,confusion	2.5x2.0x1.6	↑4.25 mmol/L	↑2300 ng/L	surgery	Emergency operation	Intractable hypercalcemia	Parathyroid adenoma with intratumoral hemorrhage
Johnston (22)	19	M	tetany, neck	3.5x2.5	↓5.1 mg. per 100 ml.	N/A	surgical exploration	N/A	N/A	The capsule surrounding the adenoma was intact and the central area was necrotic, with a rim of viable “Wasserhelle” cells around the periphery.
Novodvorsky (24)	51	M	Asymptomatic	1.7 ×0.5 × 1.0	→2.43 mmol/L	↑8.7 pmol/L	uneventful bilateral neck exploration	20M	spontaneous remission, but recurrence in 17 months	parathyroid autoinfarction
Cetani (27)	39	F	neck pain, swelling, tenderness	2.5	→1.23mmol/L	→40ng/L	parathyroidectomy	11M	spontaneous remission, but recurrence in 11months	parathyroid adenoma
Lucas (29)	67	M	Chronic symptomatic hypercalcemia	1.5x1.0	→9.9mg/dl	↑67pg/ml	Neck exploration	N/A	Spontaneous remission	Spontaneous infarction of a parathyroid adenoma
Pereira (30)	24	F	hand muscle contraction, Chvostek's sign	7.4 cm3 (Volume)	↓ 6.8 mg/dl	↑110 pg/ml	bilateral neck exploration	8Y	spontaneous remission, but recurrence in 7 years	benign proliferation of parathyroid cells
Daniel (31)	51	F	intermittent hoarseness, breathy voice, coughing, right vocal fold paresis	3.4	10.8 mg/dL	118pg/mL	minimally invasive parathyroidectomy	N/A	N/A	hypercellular parathyroid tissue
Taguchi (33)	85	F	neck mass and symptomatic hypercalcemia	3.6	↑11.7 mg/dl	↑1348pg/ml	surgery	N	N/A	cystic parathyroid adenoma with intracystic hemorrhage
Maxwell (36)	63	F	neck pain and swelling, difficulty swallowing liquids, choking sensation, voice change	4	→22pg/mL	↑10.4 mg/dL	parathyroidectomy and thyroid lobectomy,	1M	Spontaneous remission. but recurrence in 2 weeks	hypercellular parathyroid adenoma, focus of inflammation, cystic change, fibrosis, and hemosiderin deposition
Kataoka (38)	52	F	Abrupt neck pain and swelling, dysphagia, fever,mass	3.1×2.3×1.8	↓8.4 mg/dl	→40.8pg/ml^‡^	parathyroid surgery	6Y	Incomplete remission, and recurrence in 4 months	parathyroid adenoma with oxyphilic cells surrounded by normal rims
Chen (37)	82	M	abrupt thyroid enlargement and neck mass, hoarseness, trachea compression and displacement	6.9x5.2x4.8	↑1.43mmol/L	↑210.4pg/mL	surgery	N/A	N/A	cystic ectopic intrathyroidal parathyroid adenoma
Natsui (41)	59	M	symptoms of symptomatic hypercalcemia subsided. mass	3.4x2.1	→8.6mg/dl	86pg/dl^‡^	Nodulectomy	1M	Spontaneous remission	Parathyroid adenoma (erative or necrotic tissues with the hemosiderin deposition)
Ozaki (42)	64	M	symptomatic hypercalcemia, left thyroid lobe nodule	4.0x2.5x1.0	→	N/A	cervical exploration	3M	Spontaneous remission	cystic degeneration of parathyroid adenoma.
Gooding (43)	58	M	N/A	2.7×3	12mg/dl	↑129 pg/mL	surgery	N/A	N/A	hemorrhagic parathyroid cyst.
Ben-Shlomo (44)	59	M	neck pain, acute hoarseness, Abrupt dysphonia, mass, complete paralysis of the right vocal cord.	2x3	→	N/A	surgery	N/A	N/A	hyperplastic parathyroid tissue embedded in blood
Ahadizadeh (45)	55	F	neck swelling, dysphonia.	4	11.1 mg/dL	467 pg/mL	parathyroid exploration, Left hemithyroidectomy	N/A	Spontaneous remission	parathyroid adenoma with infarction
McLatchie (46)	51	M	renal colic.renal pelvic calculus	2	→	→	neck exploration	N/A	Spontaneous remission	infarcted chief cell adenoma
Efremidou (47)	59	M	abrupt neck pain,	2.2×1.8×2.9	↑12.9 mg/dl	↑146.5 pg/ml	cervical exploration and thyroidectomy	2M	Incomplete remission	parathyroid adenoma cells surrounding a central region of cystic degeneration
Govindaraj (48)	46	F	Neck mass, throat pain, chronic symptomatic hypercalcemia	2 × 1.2 × 2	→	533 pg/mL,	left neck exploration	N/A	Spontaneous remission	infarcted parathyroid gland with neovascularizing granulation tissue

*total calcium; PTH, parathyroid hormone; ^‡^iPTH, intact parathyroid hormone assay; ↓Below normal; ↑Above normal; →Within the normal range.

As shown by the literature review, conservative observation may be feasible for those achieving spontaneous remission of hyperparathyroidism after intracystic hemorrhage of a parathyroid adenoma. Given the probability of relapse, the parathyroid function and hematoma changes should be closely monitored. Surgical treatment is recommended if hyperparathyroidism recurs. Those failing to achieve remission of hyperparathyroidism after the hemorrhage can be treated by elective surgery. The choice of surgical timing should be made based on parathyroid function, health economics considerations, and the patient’s will.

## Conclusion

We present a case of a 47-year-old male patient with primary hyperparathyroidism and a hypercalcemic crisis, and hypercalcemia spontaneously relieved thereafter. Pathology results indicated an intracystic hemorrhage of bilateral parathyroid adenomas. The mismatch between tumor rapid growth and blood supply might result in infarction of parathyroid adenoma and hemorrhage. The clinical manifestations of bleeding parathyroid adenomas and the accompanying changes in parathyroid function are diverse, because of the diversity of speed and volume of hemorrhage, as well as the position of the parathyroid gland. Fine-needle aspiration biopsy of parathyroid adenoma hematoma is of limited diagnostic value and carries some risk. After literature review, it is believed that there is no consensus on the timing of surgery for intracapsular hemorrhage in primary *in situ* parathyroid adenomas and that a comprehensive assessment should be made based on the Clinical manifestations, changes in parathyroid function and the patient’s wishes. We will continue to collect such case reports and keep the literature review updated to provide the best evidence for clinical decision making.

## Data Availability Statement

The original contributions presented in the study are included in the article/supplementary material. Further inquiries can be directed to the corresponding authors.

## Ethics Statement

The studies involving human participants were reviewed and approved by Medical Ethics Committee of West China Hospital, Sichuan University. The patients/participants provided their written informed consent to participate in this study. Written informed consent was obtained from the individual(s) for the publication of any potentially identifiable images or data included in this article.

## Author Contributions

YL was involved in study concept, study design, and manuscript preparation. JL carried out the definition of intellectual content and manuscript review. HL handled data analysis and statistical analysis. HY carried out data acquisition. JQ and TWe conducted the clinical studies. TWa was involved in the literature research and manuscript editing. YY performed the role of guarantor for the integrity of the entire study. All authors contributed to the article and approved the submitted version.

## Funding

This article was supported by the National Natural Science Foundation of China (No 81701888); Science and Technology Program of Sichuan (2019YFS0239); and Health and Family Planning Commission Foundation of Sichuan Province (No. 17PJ262).

## Conflict of Interest

The authors declare that the research was conducted in the absence of any commercial or financial relationships that could be construed as a potential conflict of interest.

## Publisher’s Note

All claims expressed in this article are solely those of the authors and do not necessarily represent those of their affiliated organizations, or those of the publisher, the editors and the reviewers. Any product that may be evaluated in this article, or claim that may be made by its manufacturer, is not guaranteed or endorsed by the publisher.

## References

[1] Kozlow W Demeure MJ Welniak LM Shaker JL. Acute Extracapsular Parathyroid Hemorrhage: Case Report and Review of the Literature. Endocr Pract (2001) 7(1):32–6. doi: 10.4158/EP.7.1.32 11250766

[2] Wootten Christopher T Orzeck Eric A. Spontaneous Remission of Primary Hyperparathyroidism: A Case Report and Meta-Analysis of the Literature. Head Neck (2006) 28(1):81–8. doi: 10.1002/hed.20316 16284975

[3] Chinese Medical Association, Osteoporosis and Bone Mineral Diseases Branch Metabolic Bone Diseases Group of Endocrine Branch. Guidelines for the Diagnosis and Treatment of Primary Hyperparathyroidism. Chin J Osteoporosis Bone Mineral Dis (2014) 7(3):1–60.

[4] van den Broek JJ Poelman MM Wiarda BM Bonjer HJ Houdijk AP. Extensive Cervicomediastinal Hematoma Due to Spontaneous Hemorrhage of a Parathyroid Adenoma: A Case Report. J Surg Case Rep (2015) 2015(5):1–3. doi: 10.1093/jscr/rjv039 PMC4417131 25935903

[5] Khan S Choe CC Shabaik A. Parathyroid Adenoma Presenting With Spontaneous Cervical and Anterior Mediastinal Hemorrhage: A Case Report. Med (Baltimore) (2019) 98(5):e14347. doi: 10.1097/MD.0000000000014347 PMC6380857 30702621

[6] Zhao C Wang X Wei H Ma G. Parathyroid Adenoma Causing a Spontaneous Cervical and Mediastinal Massive Hematoma. Int J Clin Exp Med (2015) 8(11):21826–9. PMC4723996 26885150

[7] Howard JE Follis RH Jr Yendt ER Connor TB. Hyperparathyroidism; Case Report Illustrating Spontaneous Remission Due to Necrosis of Adenoma, and a Study of the Incidence of Necroses in Parathyroid Adenomas. J Clin Endocrinol Metab (1953) 13(8):997–1008. doi: 10.1210/jcem-13-8-997 13069604

[8] Nylen E Shah A Hall J. Spontaneous Remission of Primary Hyperparathyroidism From Parathyroid Apoplexy. J Clin Endocrinol Metab (1996) 81(4):1326–8. doi: 10.1210/jcem.81.4.8636326 8636326

[9] Baskar V Kamalakannan D Singh BM Odum J. Spontaneous Regression of Hypercalcemia in a Patient With Primary Hyperparathyroidism and Prolactinoma. J Endocrinol Invest (2004) 27(5):462–4. doi: 10.1007/BF03345292 15279080

[10] Huang J Soskos A Murad SM Krawisz BR Yale SH Urquhart AC. Spontaneous Hemorrhage of a Parathyroid Adenoma Into the Mediastinum. Endocr Pract (2012) 18(4):e57–60. doi: 10.4158/EP11329.CR 22805111

[11] Santelmo N Hirschi S Sadoun D Kambouchner M Cohen R Valeyre D. Bilateral Hemothorax Revealing Mediastinal Parathyroid Adenoma. Respiration (1999) 66(2):176–8. doi: 10.1159/000029364 10202326

[12] Bürgesser MV Debernardi DM Bustos ME. Spontaneous Mediastinal Hematoma as an Initial Manifestation of Ectopic Parathyroid Cystadenoma. Arch Bronconeumol (2012) 48(5):185–6. doi: 10.1016/j.arbr.2011.12.002 22386667

[13] Devèze A Sebag F Pili S Henry JF. Parathyroid Adenoma Disclosed by a Massive Cervical Hematoma. Otolaryngol Head Neck Surg (2006) 134(4):710–2. doi: 10.1016/j.otohns.2005.03.075 16564403

[14] Akimoto T Saito O Muto S Hasegawa T Nokubi M Numata A. A Case of Thoracic Hemorrhage Due to Ectopic Parathyroid Hyperplasia With Chronic Renal Failure. Am J Kidney Dis (2005) 45(6):e109–14. doi: 10.1053/j.ajkd.2005.03.004 15957122

[15] Berry BE Carpenter PC Fulton RE Danielson GK. Mediastinal Hemorrhage From Parathyroid Adenoma Simulating Dissecting Aneurysm. Arch Surg (1974) 108(5):740–1. doi: 10.1001/archsurg.1974.01350290102019 4829792

[16] Nakajima J Takamoto S Tanaka M Takeuchi E Murakawa T Kitagawa H. Parathyroid Adenoma Manifested by Mediastinal Hemorrhage: Report of a Case. Surg Today (2002) 32(9):809–11. doi: 10.1007/s005950200155 12203060

[17] DeGroote JW. Acute Intermittent Hyperparathyroidism With Hemorrhage Into a Parathyroid Adenoma. JAMA (1969) 208(11):2160–1. doi: 10.1001/jama.1969.03160110132027 5818994

[18] Chodack P Attie JN Groder MG. Hypercalcemic Crisis Coincidental With Hemorrhage In Parathyroid Adenoma. Arch Intern Med (1965) 116:416–23. doi: 10.1001/archinte.1965.03870030096016 14325916

[19] Manouras A Toutouzas KG Markogiannakis H Lagoudianakis E Papadima A Antonakis PT. Intracystic Hemorrhage in a Mediastinal Cystic Adenoma Causing Parathyrotoxic Crisis. Head Neck (2008) 30(1):127–31. doi: 10.1002/hed.20661 17615565

[20] Mizunashi K Takaya K Sato H Mori S Abe K Furukawa Y. The Time Course of Renal Function and Bone Turnover in Parathyroid Crisis Due to Intratumoral Hemorrhage. Clin Investig (1994) 72(6):448–50. doi: 10.1007/BF00180519 7950156

[21] Ferrari F Marcocci C Cetani F. Acute Severe Primary Hyperparathyroidism: Spontaneous Remission After 2 Years Follow-Up. J Endocrinol Invest (2019) 42(2):243–4. doi: 10.1007/s40618-018-0971-4 30374853

[22] Johnston CC Schnute RB. A Case of Primary Hyperparathyroidism With Spontaneous Remission Following Infarction of the Adenoma With Development of Hypocalcemic Tetany. J Clin Endocrinol Metab (1961) 21:196–200. doi: 10.1210/jcem-21-2-196 13790366

[23] Micale SJ Kane MP Busch RS. Spontaneous Resolution of Primary Hyperparathyroidism in Parathyroid Adenoma. Case Rep Endocrinol (2012) 2012:793753. doi: 10.1155/2012/793753 23198183 PMC3502788

[24] Novodvorsky P Hussein Z Arshad MF Iqbal A Fernando M Munir A. Two Cases of Spontaneous Remission of Primary Hyperparathyroidism Due to Auto-Infarction: Different Management and Their Outcomes. Endocrinol Diabetes Metab Case Rep (2019) 2019:18–0136. doi: 10.1530/EDM-18-0136 PMC6510711 31063971

[25] Kara E Della Valle E De Vincentis S. Cured Primary Hyperparathyroidism After Fine-Needle Aspiration Biopsy-Induced Parathyroid Disappearance. Endocrinol Diabetes Metab Case Rep (2017) 2017:17–0125. doi: 10.1530/EDM-17-0125 PMC5704442 29204278

[26] Schinner S Fritzen R Schott M Willenberg HS Scherbaum WA. Spontaneous Remission of Primary Hyperparathyroidism. Exp Clin Endocrinol Diabetes (2007) 115(9):619–21. doi: 10.1055/s-2007-985358 17943699

[27] Cetani F Ambrogini E Faviana P Vitti P Berti P Pinchera A. Spontaneous Short-Term Remission of Primary Hyperparathyroidism From Infarction of a Parathyroid Adenoma. J Endocrinol Invest (2004) 27(7):687–90. doi: 10.1007/BF03347505 15505996

[28] Onoda N Miyakawa M Sato K Demura H Uchida E. Spontaneous Remission of Parathyroid Adenoma Followed With Ultrasonographic Examinations. J Clin Ultrasound (1994) 22(2):134–6. doi: 10.1002/jcu.1870220213 8132794

[29] Lucas DG Lockett MA Cole DJ. Spontaneous Infarction of a Parathyroid Adenoma: Two Case Reports and Review of the Literature. Am Surg (2002) 68(2):173–6. 11842966

[30] Pereira FA Brandão DF Elias J Paula FJ. Parathyroid Adenoma Apoplexy as a Temporary Solution of Primary Hyperparathyroidism: A Case Report. J Med Case Rep (2007) 1:139. doi: 10.1186/1752-1947-1-139 18021421 PMC2204027

[31] Givens DJ Hunt JP Bentz BG. Uncommon Presentations of Parathyroid Adenoma. Head Neck (2013) 35(9):E265–268. doi: 10.1002/hed.23124 22907766

[32] Taniguchi I Maeda T Morimoto K Miyasaka S Suda T Yamaga T. Spontaneous Retropharyngeal Hematoma of a Parathyroid Cyst: Report of a Case. Surg Today (2003) 33(5):354–7. doi: 10.1007/s005950300080 12734730

[33] Taguchi T Sugimoto T Terada Y. Cystic Parathyroid Adenoma With Intracystic Hemorrhage. Endocrine (2016) 52(2):399–400. doi: 10.1007/s12020-015-0743-2 26392005

[34] Simcic KJ McDermott MT Crawford GJ Marx WH Ownbey JL Kidd GS. Massive Extracapsular Hemorrhage From a Parathyroid Cyst. Arch Surg (1989) 124(11):1347–50. doi: 10.1001/archsurg.1989.01410110109023 2684095

[35] Shim WS Kim IK Yoo SD Kim DH. Non-Functional Parathyroid Adenoma Presenting as a Massive Cervical Hematoma: A Case Report. Clin Exp Otorhinolaryngol (2008) 1(1):46–8. doi: 10.3342/ceo.2008.1.1.46 PMC2671752 19434262

[36] Maxwell JH Giroux L Bunner J Duvvuri U. Fine-Needle Thyroid Aspiration-Induced Hemorrhage of an Unsuspected Parathyroid Adenoma Misdiagnosed as a Thyroid Nodule: Remission and Relapse of Hyperparathyroidism. Thyroid (2011) 21(7):805–8. doi: 10.1089/thy.2010.0200 21615303

[37] Chen J Ma Z Yu J. Diagnostic Pitfalls in a Cystic Ectopic Intrathyroidal Parathyroid Adenoma Mimicking a Nodular Goiter: A Care-Compliant Case Report. Med (Baltimore) (2019) 98(5):e14351. doi: 10.1097/MD.0000000000014351 PMC6380700 30702624

[38] Kataoka K Taguchi M Takeshita A Miyakawa M Takeuchi Y. Recurrence of Primary Hyperparathyroidism Following Spontaneous Remission With Intracapsular Hemorrhage of a Parathyroid Adenoma. J Bone Miner Metab (2008) 26(3):295–7. doi: 10.1007/s00774-007-0816-2 18470672

[39] Kovacs KA Gay JD. Remission of Primary Hyperparathyroidism Due to Spontaneous Infarction of a Parathyroid Adenoma. Case Report and Review of the Literature. Med (Baltimore) (1998) 77(6):398–402. doi: 10.1097/00005792-199811000-00006 9854603

[40] Chan WB Chow CC King AD. Spontaneous Necrosis of Parathyroid Adenoma: Biochemical and Imaging Follow-Up for Two Years. Postgrad Med J (2000) 76(892):96–8. doi: 10.1136/pmj.76.892.96 PMC1741496 10644387

[41] Natsui K Tanaka K Suda M. Spontaneous Remission of Primary Hyperparathyroidism Due to Hemorrhagic Infarction in the Parathyroid Adenoma. Intern Med (1996) 35(8):646–9. doi: 10.2169/internalmedicine.35.646 8894740

[42] Ozaki O Sakamoto M Matsui Y. Spontaneous Remission of Hypercalcemia in a Functioning Parathyroid Cyst. Jpn J Surg (1984) 14(4):315–9. doi: 10.1007/BF02469648 6492506

[43] Gooding GA Duh QY. Primary Hyperparathyroidism: Functioning Hemorrhagic Parathyroid Cyst. J Clin Ultrasound (1997) 25(2):82–4. doi: 10.1002/(SICI)1097-0096(199702)25:2<82::AID-JCU6>3.0.CO;2-F 9023696

[44] Ben-Shlomo I Zohar S Turani H Cozacov C. Sudden Dysphonia Due to Parathyroid Apoplexy: A Rare Case of Recurrent Laryngeal Nerve Palsy. Head Neck (1990) 12(4):355–6. doi: 10.1002/hed.2880120415 2361867

[45] Ahadizadeh EN Manzoor NF Wasman J Lavertu P. Spontaneous Resolution of Hypercalcemia. Am J Otolaryngol (2017) 38(4):496–7. doi: 10.1016/j.amjoto.2017.03.003 28483147

[46] McLatchie GR Morris EW Forrester A Fogelman I. Autoparathyroidectomy: A Case Report. Br J Surg (1979) 66(8):552–3. doi: 10.1002/bjs.1800660810 486914

[47] Efremidou EI Papageorgiou MS Pavlidou E. Parathyroid Apoplexy, the Explanation of Spontaneous Remission of Primary Hyperparathyroidism: A Case Report. Cases J (2009) 2:6399. doi: 10.1186/1757-1626-2-6399 20184676 PMC2827073

[48] Govindaraj S Wasserman J Rezaee R Manolas KJ Liratzopoulos N. Parathyroid Adenoma Autoinfarction: A Report of a Case. Head Neck (2003) 25(8):695–9. doi: 10.1002/hed.10244 12884353

